# Disease-associated mitochondrial mutations and the evolution of primate mitogenomes

**DOI:** 10.1371/journal.pone.0177403

**Published:** 2017-05-16

**Authors:** William Corrêa Tavares, Héctor N. Seuánez

**Affiliations:** 1 Programa de Genética, Instituto Nacional de Câncer, Rio de Janeiro, Rio de Janeiro, Brazil; 2 Departamento de Genética, Instituto de Biologia, Universidade Federal do Rio de Janeiro, Rio de Janeiro, Rio de Janeiro, Brazil; 3 Departamento de Zoologia, Instituto de Biologia, Universidade Federal do Rio de Janeiro, Rio de Janeiro, Rio de Janeiro, Brazil; University of Florence, ITALY

## Abstract

Several human diseases have been associated with mutations in mitochondrial genes comprising a set of confirmed and reported mutations according to the MITOMAP database. An analysis of complete mitogenomes across 139 primate species showed that most confirmed disease-associated mutations occurred in aligned codon positions and gene regions under strong purifying selection resulting in a strong evolutionary conservation. Only two confirmed variants (7.1%), coding for the same amino acids accounting for severe human diseases, were identified without apparent pathogenicity in non-human primates, like the closely related Bornean orangutan. Conversely, reported disease-associated mutations were not especially concentrated in conserved codon positions, and a large fraction of them occurred in highly variable ones. Additionally, 88 (45.8%) of reported mutations showed similar variants in several non-human primates and some of them have been present in extinct species of the genus *Homo*. Considering that recurrent mutations leading to persistent variants throughout the evolutionary diversification of primates are less likely to be severely damaging to fitness, we suggest that these 88 mutations are less likely to be pathogenic. Conversely, 69 (35.9%) of reported disease-associated mutations occurred in extremely conserved aligned codon positions which makes them more likely to damage the primate mitochondrial physiology.

## Introduction

The mammalian mitogenome comprises a set of 13 genes coding for proteins of the electron transport chain, two genes specifying for 12S- and 16S rRNAs, 22 for tRNAs, and a non-coding D-loop region, altogether encompassing approximately 16,600 bp [1]. The mt-proteins of the electron transport chain together with other 80 proteins encoded by nuclear genes [2] comprise the oxidative phosphorylation (OXPHOS) machinery consisting of five multimeric protein complexes, one exclusively containing nuclear proteins (complex II) and four other (I, III, IV and V) with nuclear and mitochondrial proteins [3].

As OXPHOS dysfunction accounts for serious impairments in energy production, mt-genes have been subject to strong evolutionary constraints and a drastically restricted variability under the effect of strong negative selection [4–6] despite punctual cases of positive selection and molecular adaptations to extreme metabolic demands [7–11].

Most pathogenic mt-mutations are eliminated or maintained with significantly reduced frequencies in populations [5,6,12]. Analyses of a large set of human mitochondrial genomes showed that amino acid variants with predicted high pathogenicity were rarely detected, while those with a predicted low pathogenicity emerged several times during human evolutionary diversification [13]. Several mt-mutations are associated with metabolic disorders, human diseases or syndromes, like Leber Hereditary Optic Neuropathy (LHON), Mitochondrial Encephalomyopathy, Lactic Acidosis, and Stroke-like episodes (MELAS), Exercise Intolerance (EXIT), Mitochondrial Myopathy, Dystonia, Leigh Disease (LD), Alzheimer's Disease, Parkinson’s Disease, and Progressive Encephalomyopathy [14–18]. However, only a fraction of these mutations has been confirmed to be etiologically pathogenic by independent clinical studies, robust statistical analyses and well-known biochemical effects while most mutations are only reported to be associated with human pathologies without a clear causal relation with disease [17].

A phylogenetic approach might be useful for identifying mt-mutations etiologically responsible for OXPHOS damage because protein regions under strong functional and structural constraints are highly conserved [19]. In fact, some methods designed for predicting the pathogenicity and severity of mutations consider evolutionary conservation of amino acid residues, but usually include a large set of distantly related organisms [20,21], likely with disparate physiological demands. On the other hand, we would expect that mutations similar to those associated with pathologic conditions in humans would also reduce fitness in very closely related species, being therefore subject to purifying selection [12]. However, this would not be necessarily true if the physiological characteristics of other species were unaffected by these mutations or other compensatory mutations nullified their pathogenic effects [22]. Conversely, non-deleterious mutations might have subsisted under neutral selection, with a high probability of being maintained across different taxa and, consequently to the lack of severe constraints, higher rates of amino acid substitutions would be expected. In this scenario, mapping the occurrence of disease-associated mutations in a phylogenetic tree of closely related species may shed light on the evolutionary constraints affecting these genes [2,3,22] as well as assessing their effect as causal factors.

In this paper, we analyze the diversity of the mitogenome in 139 primate species to test whether disease-associated mutations in humans were predominantly concentrated in codons under strong evolutionary constraints and to trace these changes within a phylogenetic approach, including ancestry, number of independent events and polarity with respect to their presumed or confirmed pathogenicity in humans.

## Material and methods

### Primate phylogeny based on mitogenomes

One hundred and forty-eight reference sequences of complete mitogenomes of 139 primate species were downloaded from GenBank (S1 Table). Taxonomic sampling included all living primate families, all hominid living species, three fossil hominids (*Homo heidelbergensis*, *Homo neanderthalensis* and the unnamed *Homo* from Denisova) and two fossil lemurs (*Palaeopropithecus ingens* and *Megaladapis edwardsi*).

Separate alignments of the 13 protein-coding *mt*-genes were run with Muscle [23] and checked manually with Mega v.6.0 [24]. These alignments were subsequently concatenated in a single dataset with 11,406 bp for Maximum Likelihood (ML) phylogenetic reconstructions. ML was independently performed with PhyML v.3.1 in SeaView v.4.5.4 [25,26] and the GARLI online platform [27], with estimations of approximate likelihood-ratio tests (aLRT) using the General Time Reversible model with gamma distribution and invariable sites (GTR+Γ+I), which was indicated as the best evolutionary model by ModelGenerator v.0.85 [28]. The GTR+Γ+I model assumes a symmetric substitution matrix, in which each pair of nucleotide substitutions has a different rate, nucleotides occurring at different frequencies, rates varying among sites according a gamma distribution and some sites remaining unchanged [29]. The alignment of the 13 concatenated genes is available in S1 File.

### Analyses of evolutionary conservation of codons and amino acid residues

The evolutionary conservation of each aligned codon position and the amino acid residue therein encoded was estimated with three different indices, named Ind1, Ind2, Ind3. The simplest index (Ind1) recorded the number of different amino acids per aligned codon position. Ind2 estimated the total number of missense mutations per aligned codon position along primate radiation using SLAC in DataMonkey server [30,31]. Finally, Ind3 reported the standardized evolutionary rates per aligned residue position with ConSurf server [32,33] using a Rate4Site algorithm under an empirical Bayesian methodology [34,35]. The Rate4Site algorithm uses the tree topology and branch lengths to calculate rates of amino acid substitutions per site. The resulting conservation scores are usually highly correlated with dN/dS (ratio of non-synonymous substitutions per non-synonymous sites to synonymous substitutions per synonymous sites) scores based on the coding DNA sequences [35].

Additionally, TreeSAAP was used for investigating whether, along primate diversification, non-synonymous substitutions in codon positions with confirmed and reported disease-associated mutations resulted in substitutions of amino acids with radically different physicochemical properties [36,37]. Analysis was carried out testing 31 physicochemical amino acid properties, and only changes of categories 6, 7 and 8 with z-scores > 3.09 (p < 0.001) were considered to be radical changes [36,37]. With TreeSAAP we estimated, for each residue position, the number of independent amino-acid substitutions resulting in changes of radical physicochemical properties, and the maximum number of physicochemical properties radically affected in each residue position. Amino acid substitutions resulting in radically different physicochemical properties would be expected to occur very rarely in residue positions under strong functional constraints while substitutions by amino acids with similar properties would be expected to be frequent.

SLAC was used for identifying codons under negative selection. SLAC reconstructs codon ancestral sequences, estimates the normalized expected and observed numbers of synonymous and non-synonymous substitutions and computes dN/dS ratios. A p-value derived from a two-tailed extended binomial distribution is used to assess significance of dN and dS differences (α = 0.05). Codons are considered to be negatively selected when dN < dS.

### Disease-associated mutations per aligned codon position

The disease-associated missense mutations found in 13 protein-coding, human mt-genes were downloaded from UniProt and MITOMAP databases in November 2016 [17,38,39] for analyzing Ind1, Ind2 and Ind3 data. Two kinds of disease-associated mutations were considered according to MITOMAP criteria: (i) “reported” (when one or more publications considered a mutation as possibly pathogenic) and (ii) “confirmed” (when two or more independent laboratories reported the pathogenicity of a specific mutation, and the mitochondrial research community generally accepted it as being pathogenic). This classification will be used throughout this report.

The frequency distribution of each index (Ind1, Ind2 and Ind3) for (i) all aligned codon positions, (ii) aligned codon positions with reported disease-associated mutations, and (iii) aligned codon positions with confirmed disease-associated mutations was plotted to test whether disease-associated mutations were predominantly concentrated in highly conserved positions. The Kolmogorov-Smirnov tests were used for comparing the distributions of i-, ii- and iii- groups of aligned codon positions per index.

A t-test was used for investigating whether predicted pathogenicity scores of confirmed disease-associated mutations, estimated with MutPred [21] in a previous study [13], were higher than for reported disease-associated mutations.

## Results

### Phylogenetic reconstruction

ML analyses showed a very similar phylogenetic topology to previous reports [7,40–42] with high node support. A detailed description of this topology is available in S1 Fig. A newick formatted tree is available in S2 File.

### Variation and evolution of primate mt-genes

**Fig 1:**
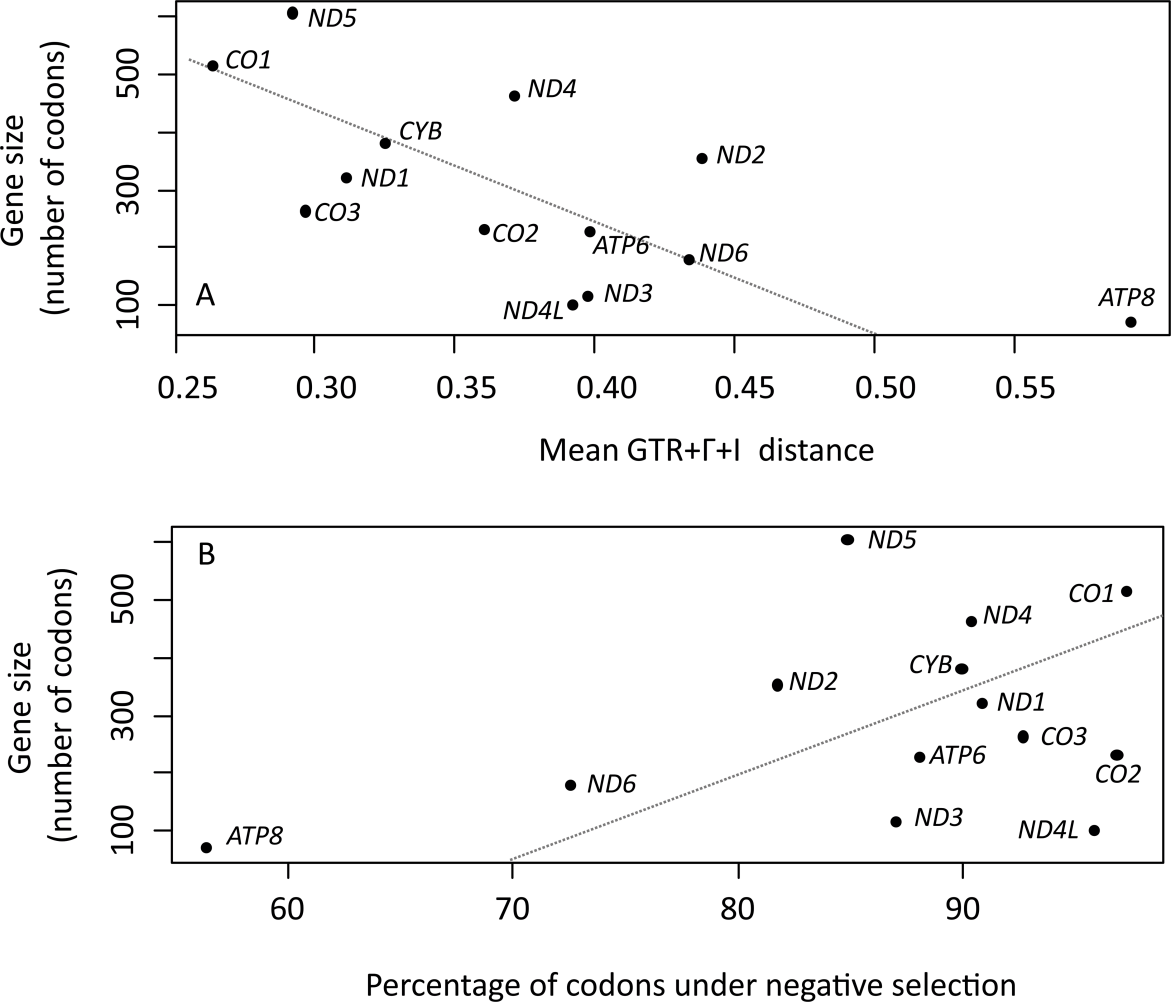
Gene size, genetic distance and codons under negative selection. (A) Significant, positive correlation between mitochondrial gene size and mean GTR+Γ+I distance (r^2^ = 0. 461; p = 0.011). (B) Non-significant, negative correlation between gene size and percentage of codons under negative selection (r^2^ = 0.136; p = 0.214).

The mt-*CO1* gene was the most evolutionarily conserved of all 13 mt-genes (mean GTR+Γ+I distance = 0.253) while mt*-ATP8* was the most variable (mean GTR+Γ+I distance = 0.582). A significant, negative correlation was observed between gene size and mean GTR+Γ+I distance (Fig 1A; r^2^ = 0.461; p = 0.011) but not between gene size and proportion of codons under negative selection (Fig 1B; r^2^ = 0.136; p = 0.214).

Except for mt-*ATP6*, mt-*CO1*, mt-*CO2*, mt-*CO3* and mt-*ND4L*, where indels were absent, 45 indels occurring as independent evolutionary events were clustered in highly variable gene regions (S2 Table). This was the case of codon 85 of mt-*ND3* with one deletion in bushbabies (*Galago*) and three independent insertions between codon 85 and 86 in mouse lemurs (*Microcebus*) and baboons (*Papio*). The mt-*ND4* gene showed four independent deletions of codon 47 in the New World monkeys (Platyrrhini), bushbabies (*Galago*), lorises (*Loris*) and gibbons (*Nomascus*), as well as two independent deletions of the adjacent codons 49 and 50 in aye-aye (*Daubentonia madagascariensis*) and lineage including galagos and lorises (Lorisiformes). Twelve independent deletions and eight independent insertions occurred between codons 105 and 115 of the mt-*ND6* gene in 20 taxa.

Analyses of 3,786 aligned codon positions showed that the number of different amino acids coded per position (Ind1) varied from 1 to 14 (mode = 1; mean = 3.020; S.D. = 2.408; Fig 1) while the number of non-synonymous substitutions per aligned codon position (Ind2) varied from 0 to 76 (mode = 0; mean = 6.564; S.D. = 9,318). The evolutionary rate of each amino acid position (Ind3) varied from − 0.719 to 6.529 (mode = − 0.707; median = − 0.479; mean of standardized rates = 0; S.D. = 1). The distributions of Ind1, Ind2 and the transformed Ind3 (for including only positive estimates) fitted well to the exponential distributions for Ind1 (χ^2^ = 272.931, df = 14, p < 0.001), Ind2 (χ^2^ = 711.737; df = 7; p < 0.001) and Ind3 (χ^2^ = 591.790; df = 8; p < 0.001). In 1,443 (38.1%) of aligned codon positions, missense substitutions were not observed across primate diversification because only a single amino acid was coded in all species. On the other hand, 3,381 (88.9%) aligned codon positions appeared to be under negative selection with SLAC.

### Aligned codon positions and disease-associated mutations in humans

**Fig 2:**
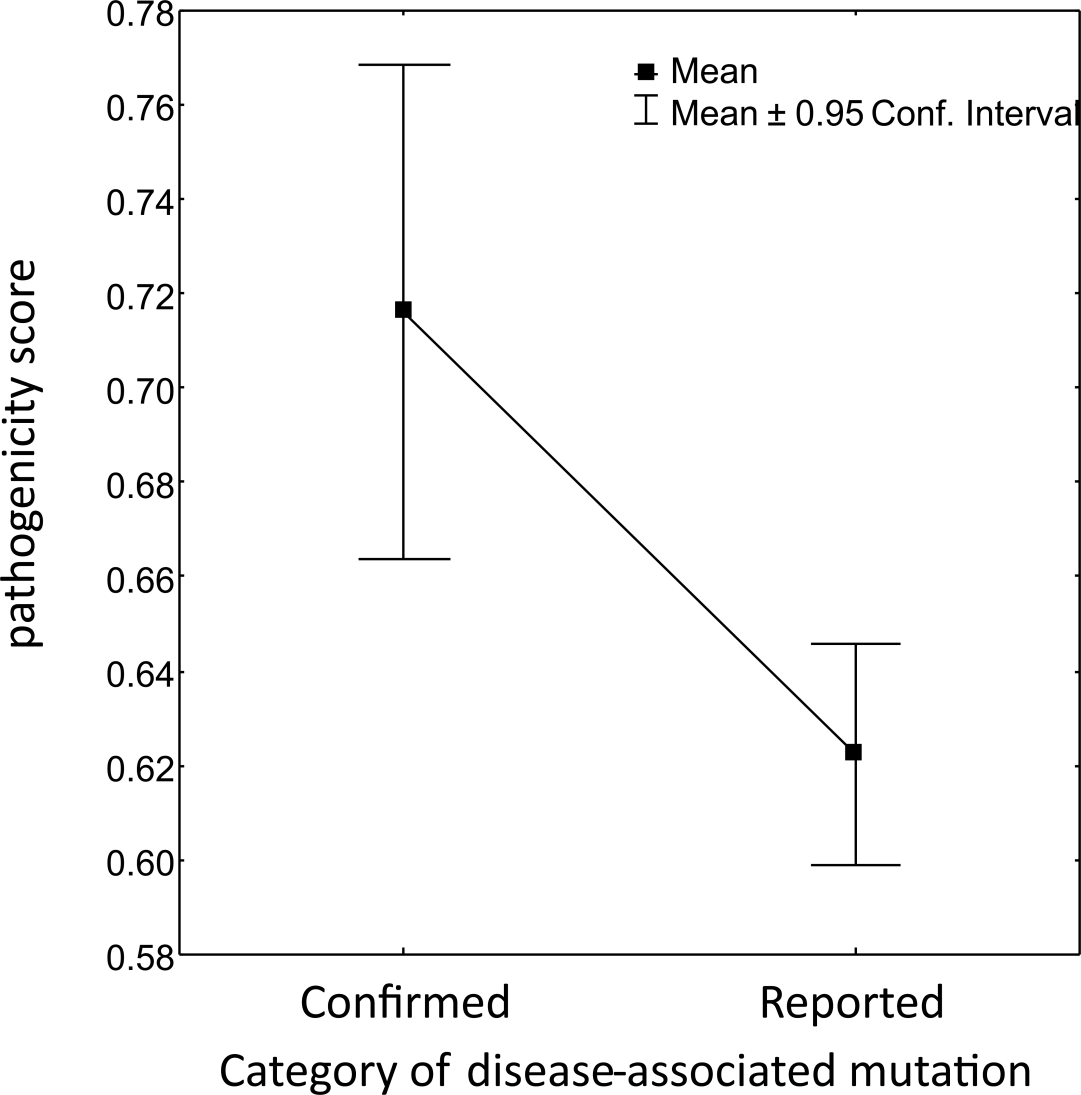
Predicted pathogenicity scores. Significant difference between means of predicted pathogenicity scores between confirmed disease-associated mutations and reported disease-associated mutations (t = 2.956; d.f. = 226; p = 0.003).

**Fig 3:**
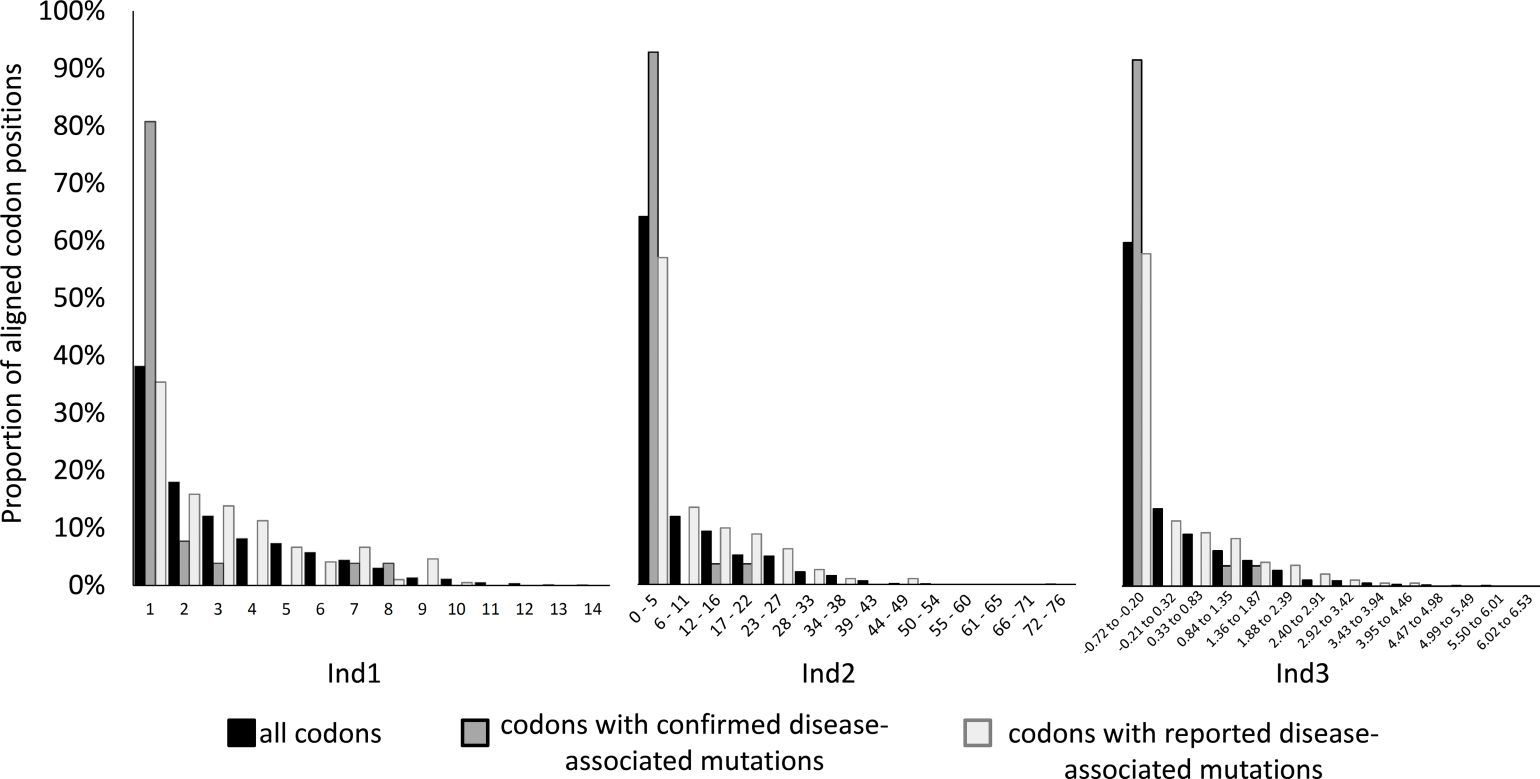
Indices of evolutionary conservation. Frequency distributions of Ind 1 (left), Ind2 (middle) Ind3 (right) of three groups of aligned codon positions (all positions, positions with confirmed disease-associated mutations and positions with reported disease-associated mutations).

Missense substitutions associated with human diseases were identified in 220 aligned codon positions (S3 Table). Twenty-eight aligned codon positions showed mutations classified as “confirmed” and 192 as “reported” by MITOMAP and UNIPROT. The predicted pathogenicity scores of the confirmed disease-associated mutations were significantly higher than those of reported disease-associated mutations (t = 2.956; d.f. = 226; p = 0.003; Fig 2). Kolmogorov-Smirnov tests of Ind1, Ind2 and Ind3 consistently showed that aligned codon positions with confirmed disease-associated mutations were not randomly distributed but concentrated among the most evolutionary conserved positions. On the other hand, Ind1, Ind2 and Ind3 estimates showed that the aligned codon positions with reported disease-associated mutations were not significantly concentrated and were not significantly different from the whole set of aligned codon positions (Fig 3; Table 1). All aligned codon positions with confirmed disease-associated mutations were found to be under negative selection with SLAC, contrary to only 85.5% of aligned codon positions with reported disease-associated mutations, similarly to found for all positions of all mt-genes (88.9%).

**Table 1:** Comparisons of Ind1, Ind2 and Ind2 distributions.

	Ind1			Ind2			Ind3		
	All codons	Confirmed	Reported	All codons	Confirmed	Reported	All codons	Confirmed	Reported
**All codons**	-	4.3E-04	0.888	-	2.6E-04	0.1724	-	4.1E-05	0.666
**Confirmed**	0.397	-	4.6 E-04	0.409	-	4.6E-04	0.457	-	1.0E-04
**Reported**	0.043	0.420	-	0.0817	0.420	-	0.054	0.465	-

Kolmogorov-Smirnov test for comparing distributions of estimates Ind1, Ind2 and Ind2 between sets of all aligned codon positions (All codons), aligned codon positions with confirmed disease-associated mutations (Confirmed), and aligned codon positions with reported disease-associated mutations (Reported). The *D* statistics of each test are shown above diagonals and respective *p*-values are shown below diagonals.

### Aligned codon positions with confirmed disease-associated mutations in humans

Of the 28 aligned codon positions with confirmed disease-associated mutations, 78.6% (n = 22) were invariant in all reference sequences of the primate species herein analyzed. Of the six positions with missense mutations, two were found to be highly variable across primate taxa. This was the case of codon positions 34 and 45 of mt-*ND3* with missense substitutions m.10158T>C (S34P) and m.10191T>C (S45P) accounting for confirmed disease-associated mutations with Leigh Disease in humans. In these two positions, serine is the only coded residue in normal humans. These positions, however, were found to be highly variable along primate evolution (Ind3_34_ = 1.442; Ind3_45_ = 0.882), with 20 and 15 non-synonymous substitutions, coding for eight and six amino acids, respectively, albeit none encoding proline. Among the 20 non-synonymous substitutions affecting codon 34 of mt-*ND3*, 10 resulted in radical changes in amino acid physicochemical properties (affecting up to seven different properties), and among the 15 non-synonymous substitutions affecting codon 45 of mt-*ND3*, six resulted in radical changes in amino acid physicochemical properties (affecting up to 12 different properties).

**Fig 4:**
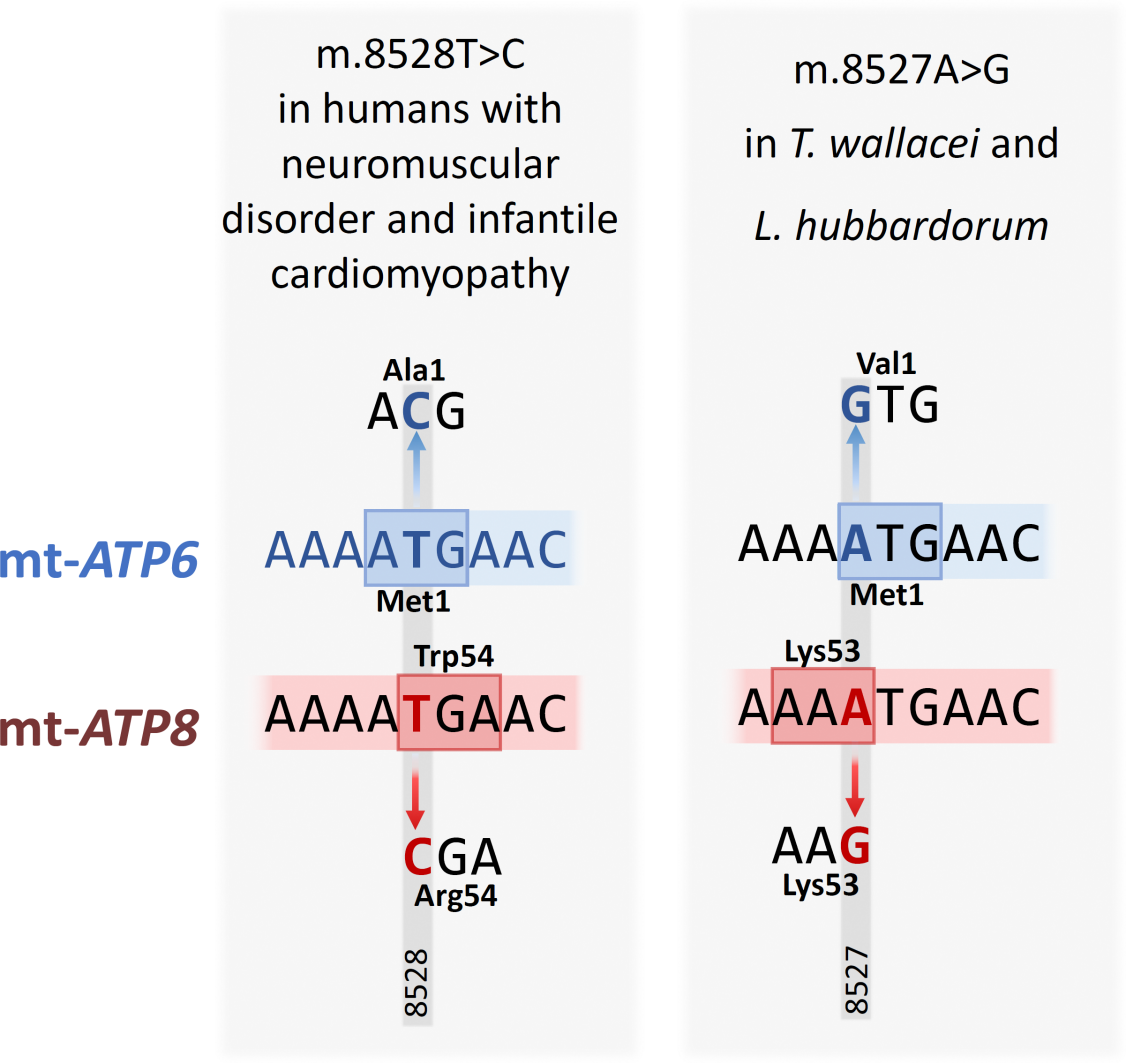
Overlapping mutations in mt-*ATP6* and mt-*ATP8*. Comparison between a confirmed disease-associated missense mutation in humans (m.8528T>C; left) and a mutation in an adjacent region (m.8527A>G, right) in two non-human primates, *Tarsius wallacei* and *Lepilemur hubbardorum*. These mutations affected nucleotide positions in overlapping coding regions of mt-*ATP6* (in blue, above) and mt-*ATP8* (in red, below). m.8528T>C is a missense mutation in both genes, while m.8527A>G is a missense mutation only in mt-*ATP6*.

Confirmed disease-associated mutations in humans were found to occupy two slightly variable aligned codon positions, but with amino acid physicochemical properties highly conserved along primate evolution. This was the case of the m.8528T>C substitution in the partially overlapping codons 1 of mt-*ATP6* and 54 of mt-*ATP8* resulting in M1T and W54R, respectively, and associated with neuromuscular disorder and infantile cardiomyopathy (Fig 4). Along the primate diversification, the amino acid substitutions in these residue positions did not result in any radical change in physicochemical properties. A phylogenetic reconstruction indicated that ATG was the ancestral codon 1 of mt-*ATP6* and TGA the ancestral codon of codon 54 of mt-*ATP8*. Two independent m.8527A>G substitutions resulted in identical amino acid replacements (M1V) in the first residue of the ATP synthase F_0_-a polypeptide in the Wallace's tarsier (*Tarsius wallacei*) and Hubbard's sportive lemur (*Lepilemur hubbardorum*), while m.8527A>G in codon 53 of mt-*ATP8* was a samesense mutation.

Moreover, in the aligned codon position 64 of mt-*ND6*, three different missense mutations (m.14482C>A; M64I, m.14482C>G; M64I, and m.14484T>C; M64V) resulting in substitutions of isoleucine and valine for methionine, have been confirmed to be associated with LHON in humans. A phylogenetic reconstruction indicated that codon 64 has been variable along primate evolution, with TTG being the most ancestral triplet, coding for leucine, and with all five other leucine codons being present along different lineages. Methionine is therefore a derived trait (L64M) resulting from two different, independent missense mutations, one in the fat-tailed dwarf lemur (*Cheirogaleus medius*) lineage (CTG→ATG) and another in the ancestor of all anthropoid (simiiforme) primates (TTG→ATG). Within this latter lineage, methionine has been coded by both ATG and ATA. The two non-synonymous mutations affecting codon 64 of mt-*ND6* did not result in any radical change in physicochemical properties.

**Fig 5:**
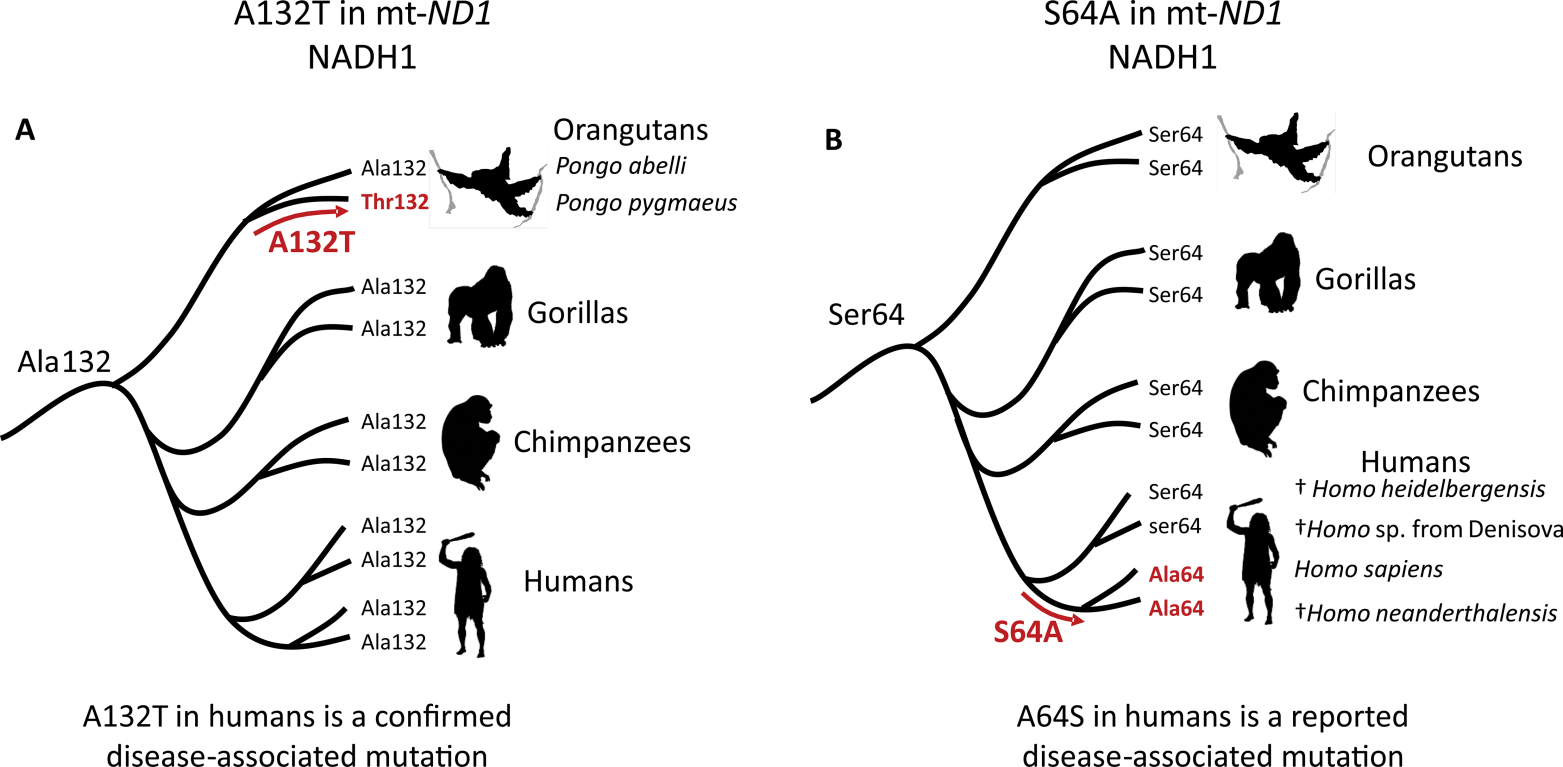
Phylogeny of the Hominidae, showing two disease-associated mutations in modern humans (*Homo sapiens*) with similar counterparts in other hominids. A. Confirmed, disease-associated A132T mutation in mt-*ND1* showing its occurrence in the Bornean orangutans (*Pongo pygmaeus*). B. Reported, disease-associated, A64S mutation in mt-*ND1*, showing that all hominids show serine in residue 64 of NADH1, except modern humans and closely related Neanderthals.

In none of the four above mentioned codon positions, specific amino acids from confirmed disease-associated mutations were found in the non-human primate species herein studied. This, however, was not the case of both mt-*ND1* missense mutations m.3635G>A (S110N) and m.3700G>A (A132T), identified as confirmed disease-associated mutations with LHON in humans. Asparagine and threonine, resulting from these mutations were deduced from the reference sequence of the sooty mangabey (*Cercocebus atys*) and the Bornean orangutan (*Pongo pygmaeus*), respectively, apparently without evidence of a pathologic condition in these species. Furthermore, in another available mt-*ND1* sequence of *C*. *atys* (KP090062) asparagine was also found at position 110, showing that this substitution was not a peculiarity of the reference specimen. Similarly, in all four other available mt-*ND1* sequences of *P*. *pygmaeus* (NC_001646, X97713.1, X97713.1 and X97713.1) threonine was coded by codon 132, while in four other sequences of the Sumatran orangutan (*Pongo abelli*; X97713.1, X97713.1, X97713.1 and X97713.1) this position coded for alanine (Fig 5A). The substitution of serine by asparagine, the only amino acid change in residue position 110 of NADH1, did not result in radical changes in physicochemical properties. Conversely, two of the three amino acid substitutions affecting residue 132 of NADH1 resulted in radical changes in physicochemical properties (affecting up to three different properties); the substitution of alanine by threonine, as observed in the linage leading to *P*. *pygmaeus*, corresponded to a radical change in the tendencies of forming alpha-helices.

### Aligned codon positions with reported disease-associated mutations in the human

Among the 192 aligned codon positions with reported disease-associated mutations in the human, only 35.9% (n = 69) did not show missense mutations, similarly to the proportion of codon positions without missense substitutions of all mt-genes (38.114%). In the other 123 aligned codon positions, 88 (71.5%) mutations were found to code for amino acids associated with human disease in at least one non-human primate species (Table 2; S3 Table and S2 Fig). This was the case of a missense mutation in mt-*CO1* codon 4 (m.5913G>A; D4N) substituting asparagine for aspartic acid, and associated with human prostate cancer and hypertension. Asparagine was estimated to be the ancestral primate residue for this position with SLAC, and was also present in 119 other primate species herein analyzed. Similarly, a missense mutation in codon 159 of mt-*ND5* (m.12811T>C; Y159H) substituting histidine for tyrosine was reported to be a likely LHON factor [43]. However, the presence of histidine in NADH5 residue position 159 independently appeared 11 times along primate diversification, in 70 different species herein analyzed.

**Table 2:** Number of aligned codon positions occupied by disease-associated mutations.

	Gene	Total number of aligned codon positions	Positions with invariable residues	Positions with variable residues	Positions with disease-associated residues in primates other than *H*.*sapiens*	Positions with disease-associated residues in catarrhines other than *H*.*sapiens*	Positions with disease-associated residues in hominids other than *H*.*sapiens*	Positions with disease-associated residues in extinct *Homo* species
**Aligned codon positions with confirmed disease-associated mutations**	mt-*ATP6*	4	3	1	0	0	0	0
	mt-*ATP8*	0	-	-	-	-	-	-
	mt-*CO1*	0	-	-	-	-	-	-
	mt-*CO2*	0	-	-	-	-	-	-
	mt-*CO3*	0	-	-	-	-	-	-
	mt-*CYB*	3	3	0	0	0	0	0
	mt-*ND1*	9	7	2	2	2	1	0
	mt-*ND2*	0	0	0	0	0	0	0
	mt-*ND3*	3	1	2	0	0	0	0
	mt-*ND4*	1	1	0	0	0	0	0
	mt-*ND4L*	1	1	0	0	0	0	0
	mt-*ND5*	3	3	0	0	0	0	0
	mt-*ND6*	4	3	1	0	0	0	0
	Total	28	22	6	2	2	1	0
**Aligned codon positions with reported disease-associated mutations**	mt-*ATP6*	19	6	13	11	7	4	0
	mt-*ATP8*	8	1	7	6	5	0	0
	mt-*CO1*	26	11	15	13	13	3	1
	mt-*CO2*	14	4	10	7	6	1	1
	mt-*CO3*	12	3	9	6	6	2	0
	mt-*CYB*	22	11	11	6	6	5	1
	mt-*ND1*	25	10	15	10	10	7	1
	mt-*ND2*	12	2	10	4	1	1	1
	mt-*ND3*	4	1	3	2	2	1	1
	mt-*ND4*	9	2	7	5	4	4	1
	mt-*ND4L*	3	2	1	1	1	0	0
	mt-*ND5*	26	12	14	11	10	7	1
	mt-*ND6*	12	5	7	6	5	1	0
	Total	192	70	122	88	76	36	8

**Fig 6:**
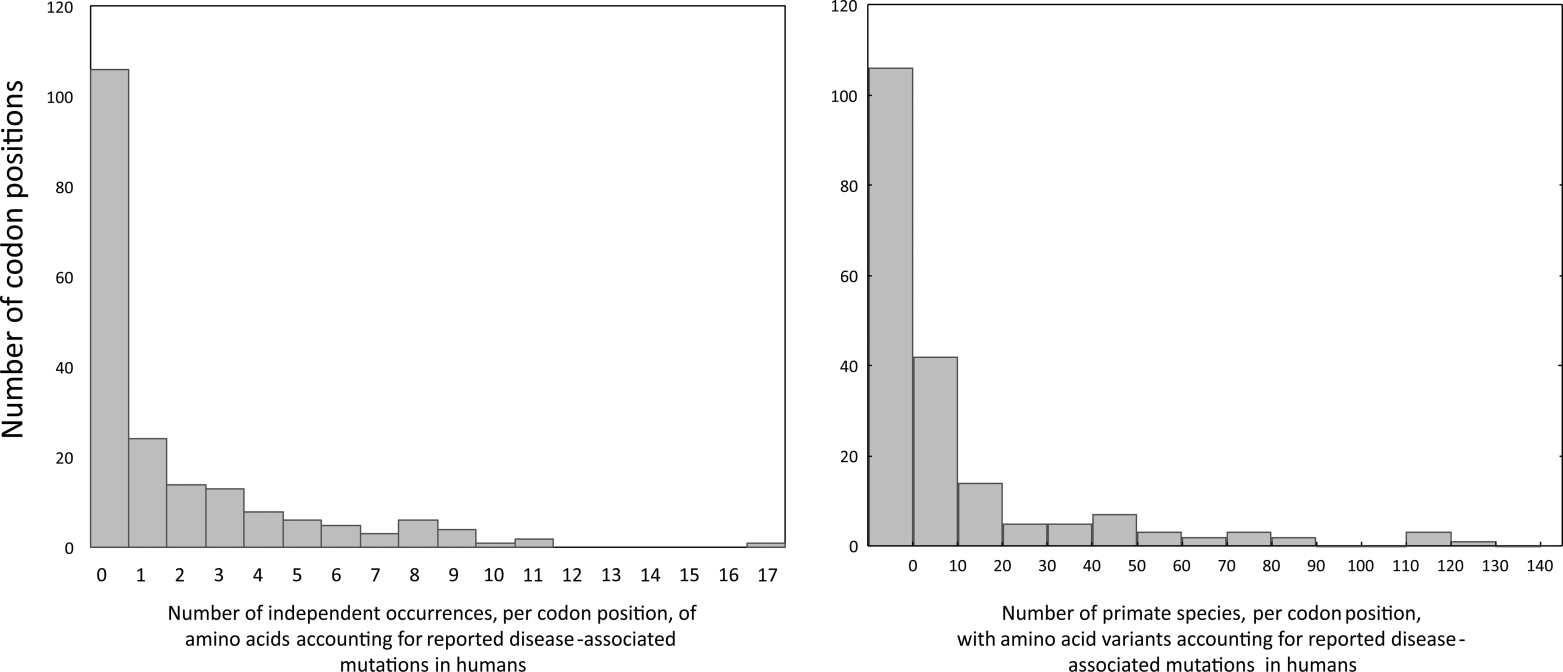
Recurrence of reported disease-associated mutations in the human across primate phylogeny. Left: Number of independent occurrences, per codon position, of amino acids accounting for reported disease-associated mutations in the human across primate phylogeny. Right: Number of species with amino acids accounting for reported disease-associated mutations in the human.

Several amino acids coded for by reported disease-associated mutations in humans were also coded for by 76 aligned codon positions in at least one Old World primate (catarrhine) species. Similarly, this was observed at 36 aligned codon positions, 24 in great ape species and eight in species of the genus *Homo* (Table 2 and S3 Table). The mean number of independent appearances, per codon position, of amino acids resulting from reported disease-associated mutations in the human equaled 1.7 (min = 0; max = 17; S.D. = 2.844), and the mean number of primate species with amino acid variants resulting from reported disease-associated mutations in the human equaled 11.1 per codon position (min = 0; max = 121; S.D. = 23.780; Fig 6).

Sixty-nine aligned codon positions where amino acid replacements resulted in reported disease-associated mutations were found to be completely conserved (with Ind1 = 1 and Ind2 = 0) and under negative selection along primate evolution (Table 3). Most of these mutations in these codons were associated with MELAS (n = 14), LHON (n = 13), Leigh Disease (n = 9), and EXIT (n = 5). Non-synonymous substitutions that did not result in radical changes in amino acid properties were found to occur in other 14 aligned codon positions where amino acid replacements resulted in reported disease-associated mutations in humans (Table 4).

**Table 3:** Reported disease-associated, missense mutations occurring in aligned codons positions with single invariant amino acid residues along primate evolution.

Gene	Nucleotide substitution	Codon position in humans	Amino acid substitution	Associated phenotype in humans
mt*-ATP6*	m.8561C>G	12	P12R	Ataxia with neuropathy, DM, SNHL and hypogonadism
mt*-ATP6*	m.8741T>G	72	L72R	MILS
mt*-ATP6*	m.8851T>C	109	W109R	BSN
mt*-ATP6*	m.8969G>A	148	S148N	MLASA
mt*-ATP6*	m.9016A>G	164	I164V	LHON
mt*-ATP6*	m.9035T>C	170	L170P	Ataxia syndromes
mt*-ATP6*	m.9191T>C	222	L222P	LD
mt*-ATP8*	m.8528T>C	55	W55R	Mitochondrial complex V deficiency
mt*-CO1*	m.5935A>G	11	N11S	PC
mt*-CO1*	m.6277G>A	125	G125D	CC
mt*-CO1*	m.6328C>T	142	S142F	EXIT
mt*-CO1*	m.6489C>A	196	L196I	Therapy-Resistant Epilepsy
mt*-CO1*	m.6597C>A	232	Q232K	MELAS-like syndrome
mt*-CO1*	m.6955G>A	351	G351D	Mild EXIT and moderate mental retardation
mt*-CO1*	m.7023G>A	374	V374M	MELAS-like syndrome
mt*-CO1*	m.7041G>A	380	V380I	PC
mt*-CO1*	m.7305A>C	468	M468L	PC
mt*-CO2*	m.7637G>A	18	E18K	PD risk factor
mt*-CO2*	m.7671T>A	29	M29K	MM
mt*-CO2*	m.7877A>C	98	K98Q	PEG
mt*-CO2*	m.7989T>C	135	L135P	Rhabdomyolysis
mt*-CO3*	m.9267G>C	21	A21P	MIDD
mt*-CO3*	m.9544G>A	113	G113E	Sporadic bilateral optic neuropathy
mt*-CO3*	m.9789T>C	195	S195P	Myopathy
mt*-CYB*	m.14841A>G	32	N32S	LHON
mt*-CYB*	m.14846G>A	34	G34S	EXIT
mt*-CYB*	m.15024G>A	93	C93Y	Possible DEAF modifier
mt*-CYB*	m.15092G>A	116	G116S	MELAS
mt*-CYB*	m.15197T>C	151	S151P	EXIT
mt*-CYB*	m.15209T>C	155	Y155H	Prader-Willi syndrome
mt*-CYB*	m.15243G>A	166	G166E	HCM
mt*-CYB*	m.15615G>A	290	G290D	EXIT, and Antimycin resistance
mt*-CYB*	m.15635T>C	297	S297P	Polyvisceral failure
mt*-CYB*	m.15699G>C	318	R318P	Muscle Weakness SNHL and Migraine
mt*-CYB*	m.15762G>A	339	G339E	MM
mt*-ND1*	m.3380G>A	25	R25Q	MELAS
mt*-ND1*	m.3388C>A	28	L28M	Maternally Inherited Nonsyndromic Deafness
mt*-ND1*	m.3418A>G	38	N38D	AMegL
mt*-ND1*	m.3481G>A	59	E59K	MELAS / Progressive Encephalomyopathy
mt*-ND1*	m.3688G>A	128	A128T	LD
mt*-ND1*	m.3946G>A	214	E214K	MELAS
mt*-ND1*	m.3949T>C	215	Y215H	MELAS
mt*-ND1*	m.3959G>A	218	G218D	MELAS
mt*-ND1*	m.3995A>G	230	N230S	MELAS
mt*-ND1*	m.4160T>C	286	M286P	LHON
mt*-ND2*	m.4633C>G	55	A55G	LHON
mt*-ND2*	m.5244G>A	259	G259S	LHON
mt*-ND3*	m.10254G>A	66	D66N	LD
mt*-ND4*	m.11232T>C	160	L160P	CPEO
mt*-ND4*	m.11375A>C	206	K206Q	sCJD
mt*-ND4L*	m.10543A>G	25	H25R	LHON
mt*-ND4L*	m.10591T>G	41	F41C	LHON
mt*-ND5*	m.12770A>G	145	E145G	MELAS
mt*-ND5*	m.12782T>G	149	I149S	LHON
mt*-ND5*	m.12848C>T	171	A171V	LHON
mt*-ND5*	m.13042G>A	236	A236T	Optic neuropathy, retinopathy, and LD
mt*-ND5*	m.13045A>C	237	M237L	MELAS, LHON, and Leigh overlap syndrome
mt*-ND5*	m.13063G>A	243	V243I	Adult-onset Encephalopathy and Ataxia
mt*-ND5*	m.13084A>T	250	S250C	MELAS, LD
mt*-ND5*	m.13094T>C	253	V253A	Ataxia with PEO, MELAS, LD, myoclonus and fatigue
mt*-ND5*	m.13379A>C	348	H348S	LHON
mt*-ND5*	m.13511A>T	392	K392M	Leigh-like syndrome
mt*-ND5*	m.13730G>A	465	G465E	LHON
mt*-ND5*	m.14091A>T	585	K585N	Developmental delay, seizure, hearing loss and diabetes
mt*-ND6*	m.14600G>A	25	P25L	LD with optic atrophy
mt*-ND6*	m.14498T>C	59	Y59C	LHON
mt*-ND6*	m.14453G>A	74	A74V	MELAS
mt*-ND6*	m.14439G>A	79	P79S	Mitochondrial Respiratory Chain Disorder
mt*-ND6*	m.14430A>G	82	W82R	TC

Note: DM: Diabetes Mellitus; AMegL: Acute Megakaryoblastic Leukemia; BSN: Bilateral Striatal Necrosis; CC: Colorectal cancer; CPEO: Chronic Progressive External Ophthalmoplegia; DEAF: Maternally inherited DEAFness or aminoglycoside-induced DEAFness; EXIT: Exercise Intolerance; HCM: Inherited Hypertrophic CardioMyopathy; LD: Leigh Disease; LHON: Leber Hereditary Optic Neuropathy; MELAS: Mitochondrial Encephalomyopathy, Lactic Acidosis, and Stroke-like episodes; MIDD: Maternally Inherited Diabetes and Deafness; MILS: Maternally Inherited Leigh Syndrome; MLASA: Mitochondrial Myopathy, Lactic Acidosis, and Sideroblastic Anemia; MM: Mitochondrial Myopathy; PC: Prostate Cancer; PD: Parkinson’s Disease; PEG: Pseudoexfoliation Glaucoma; PEO: Progressive External Ophthalmoplegia; sCJD: sporadic Creutzfeldt-Jakob disease; SNHL: Sensorineural Hearing Loss; TC: Thyroid Cancer.

**Table 4:** Reported, disease-associated missense mutations in aligned codons positions with variable amino acid residues along primate evolution resulting in amino acid changes with similar physicochemical properties according to TreeSAAP.

Gene	Nucleotide substitution	Codon position in humans	Amino acid substitution	Associated phenotype in humans
mt*-ATP6*	m.8836A>G	104	M104V	LHON
mt*-ATP6*	m.8890A>G	122	K122E	Juevnile-onset metabolic syndrome
mt*-CO1*	m.6267G>A	122	A122T	PC
mt*-CO1*	m.6285G>A	128	V128I	PC
mt*-CO1*	m.6721T>C	273	M273T	Acquired Idiopathic Sideroblastic Anemia
mt*-CO1*	m.6742T>C	280	I280T	Acquired Idiopathic Sideroblastic Anemia
mt*-CO1*	m.7080T>C	393	F393L	PC
mt*-CO2*	m.7587T>C	1	M1T	MELAS
mt*-CO3*	m.9438G>A	78	G78S	LHON
mt*-CO3*	m.9957T>C	251	F251L	PEM / MELAS / NAION
mt*-ND2*	m.4648T>C	60	F60S	PEG
mt*-ND2*	m.4852T>A	128	L128Q	LHON
mt*-ND3*	m.10086A>G	10	N10D	Hypertensive end-stage renal disease
mt*-ND5*	m.13271T>C	312	L312P	EXIT

Note: EXIT: Exercise Intolerance; LHON: Leber Hereditary Optic Neuropathy; MELAS: Mitochondrial Encephalomyopathy, Lactic Acidosis, and Stroke-like episodes; NAION: non‐arteritic ischaemic optic neuropathy; PC: Prostate Cancer; PEG: Pseudoexfoliation Glaucoma; PEM: Progressive encephalopathy.

## Discussion

### Primate phylogeny

Well-resolved phylogenies are necessary for tracing character evolution in morphological, physiological, ecological, biogeographic, behavioral and molecular studies [44]. This can be achieved by the continuous input of primate genomic data (nuclear and mitochondrial) from which more robust and reliable phylogenetic topologies can be reconstructed [45–47]. The ML topology herein provided (S1 Fig), was largely consistent with previous ones [40–42,46–53] and was valuable for mapping nucleotide and amino acid changes and assessing the evolutionary conservation of codons and amino acids.

### Evolutionary constraints on mt-genes

Our findings, based on the frequency of the dN/dS ratio, showed that a large fraction of codon positions (88.9%) had been subjected to negative selection along primate radiation, in agreement with the postulation that mt-genes evolved under a strong purifying selection [54,55]. Codons under negative selection were particularly concentrated in the highly-conserved genes of complex IV (mt-*CO1*, mt-*CO2* and mt-*CO3*) and mt-*ND4L*, while mt-*ATP8* and mt-*ND6* showed the lowest proportions of codons under negative selection. Reports on several mammalian orders, amphibians, birds and reptiles also found Complex IV and mt-*ND4L* to be highly conserved and mt-*ATP8* and mt-*ND6* as the most variable mt-genes [8,56,57]. This divergent pattern of molecular conservation and variation has been apparently constant at least from the beginning of tetrapod diversification. Furthermore, gene size and evolutionary conservation were found to be positively correlated as was the case of mt-*CO1*, in agreement with the proposition that the length of coding region of a gene affects its evolutionary rate [58–60] and is positively correlated with essentiality [61].

The four genes with the highest concentration of codons under negative selection did not show indels, while, on the other hand, *mt-ND6* showed 20 indels that took place as independent evolutionary events. The high conservation of complex IV genes and mt-*ND4L* indicated that mutations affecting these genes were likely to be more drastically adverse for fitness than mutations in other genes. In fact, complex IV genes and mt-*ND4L* did not show any mutation corresponding to a confirmed, disease-associated mutation in humans. Altogether, these findings suggested that these missense mutations have been consistently eliminated along the evolution of the non-human primates herein studied, while other missense mutations, corresponding to reported, disease-associated mutations in humans have been maintained.

### Disease-associated mt-mutations in humans within an evolutionary context in the primates

Several mt-mutations have been associated with human diseases [62] mainly with common adult forms of inherited neurological disorders [63]. Most confirmed mt-mutations affect tRNA genes and the translational efficiency of mitochondria impairing the proper functioning of four OXPHOS enzyme complexes [18]. Conversely, the effects of mt-mutations on protein-coding genes are more restricted and more difficult to be clearly identified [18] and mutations in only 28 codon positions are generally considered pathogenic by the mitochondrial research community (MITOMAP database [17]).

In this study, confirmed, disease-associated mt-mutations showed higher scores of predicted pathogenicity than reported ones and all of them were located in aligned codon positions under strong negative selection, and almost all in highly conserved positions along primate evolution (Table 3). This finding suggested that similar mutations in other primate species would be likely to be pathogenic and impair fitness. In fact, most of these 28 codons were responsible for critical regions of protein domains directly involved in OXPHOS. This was the case of the mt-*ATP6* region encoding a transmembrane domain of the ATP synthase F_0_ subunit involved in proton translocation [64]. One mutation (m.8993T>C) affecting this region has been etiologically associated with NARP, Leigh Disease, MILS and other diseases in humans [65]. Similarly, m.15579A>G, affecting one mt-*CYB* region encoding a protein segment exposed to the intermembrane space bearing an ubiquinone binding site has been etiologically associated with multisystem disorder and EXIT in humans [3,66,67].

Only two confirmed disease-associated mutations, both in mt-*ND3* (m.10158T>C, S34P; m.10191T>C, S45P), were located in highly variable codon positions, flanking a highly conserved mt-*ND3* loop domain (S3 Table and S2 Fig). Previous predictions about their pathogenicity have been contradictory [13,68] while a previous report has indicated that m.10158T>C affected an extremely conserved amino acid position among mammals [69], a finding that was not corroborated in the primates herein studied. A previous report proposed that a hydrophobic residue like proline at position 45 would disrupt folding of the NADH-ubiquinone oxidoreductase chain 3 and showed that only hydrophilic amino acids were present across different taxa in this position [70]. Missense mt-mutations, however, resulting in residues with manifold physicochemical properties, including hydrophobic amino acids other than proline have been identified in this study. It is therefore likely that mutations at this site have not been severely detrimental for most primates or, alternatively, a proline-45 residue may be specifically disruptive for folding of NADH-ubiquinone oxidoreductase chain 3.

Only two confirmed disease-associated missense mutations in humans, both in mt-*ND1* (m.3635G>A; S110N and m.3700G>A; A132T), showed similar counterparts in other primates, in apparently healthy sooty mangabeys (*Cercocebus atys*) and Bornean orangutans (*Pongo pygmaeus*), respectively [71,72]. This latter mutation was located in a highly conserved mt-*ND1* region, comprising approximately 27 codons, encoding a loop domain exposed to the intermembrane space, a critical region for NADH catalytic function [73,74]. The presence of a polar amino acid like threonine at position 132 in Bornean orangutans was unexpected, since this position appeared to be invariably occupied by alanine, a non-polar amino acid, in several mammals other than primates (mouse, rabbit, horse, cattle, whale, seal, cat and platypus), other animals (nematodes, sea-urchins, fish and chickens), and plants; the only exception being found in two fungi species with the polar amino acid serine [71]. Conversely, m.3635G>A (S110N) occurred in an adjacent region to the loop domain, encoding a slightly more variable transmembrane domain, presumably without a critical relevance for NADH function [74].

Occurrence of some pathogenic alleles for humans in healthy gorillas, chimpanzees and macaques has been documented for several nuclear genes [75,76]. It is not completely understood how these alleles might be innocuous for non-human primates; it is speculated that compensatory changes in other genome regions or environmental differences might neutralize their effects [22,76]. A third explanation, postulating the existence of different physiological demands between species, might explain why an allele responsible for low ATP production might be severely detrimental to a species with a high metabolic demand, like *Homo sapiens*, but not to species with low metabolisms like the slow lories (genus *Nycticebus*; [22]). Orangutans show an extremely low rate of energy use, even lower relative to body mass, than nearly any eutherian mammal ever studied [77]. This may explain why the S110N substitution in the NADH-ubiquinone oxidoreductase chain 1, affecting a highly conserved polypeptide domain, might be tolerated in orangutans but not in other mammals.

Interestingly, the mt-*ATP6* start codon changed from ATG (coding for methionine) to GTG (coding for valine) in two non-anthropoid (strepsirrhine) species, the Wallace’s tarsier (*Tarsius wallacei*) and the Hubbard's sportive lemur (*Lepilemur hubbardorum*). In humans, the first, GTG, codon of mt-*ATP6* does not completely impair gene transcription and translation, but is reportedly associated with LHON [78]. Unfortunately, it was not possible to access whether the GTG condition might be a common finding in these two species due to dearth of mt-*ATP6* data.

Differently from confirmed disease-associated mutations, the reported ones were not especially concentrated in highly conserved codon positions, suggesting that an important fraction of them have not effectively reduced organismal fitness along primate diversification. Additionally, they showed lower predicted pathogenicity scores than confirmed disease-associated mutations and 45% of them also showed similar substitutions in other primates, including very closely related species, like great apes and other species of the genus *Homo*. This was the case of the m.3496G>T (A64S), affecting mt-*ND1* of Japanese families and associated with LHON [79]. Codon 64 was highly variable along primate evolution, but more recently coded for serine in the ancestral hominid and all descendant hominid species except *Homo sapiens* and *H*. *neanderthalensis* in which an S64A mutation occurred. The m.3496G>T (A64S) found in Japanese families can thus be interpreted as an evolutionary reversion (Fig 5B).

In fact, all reported disease-associated mutations in humans with similar substitutions in the reference sequence of *Homo* species other than *Homo sapiens* (m.6150G>A, m.8021A>G, m.15077G>A, m.3421G>A, m.3496G>T, m.4659G>A, m.10398A>G, m.11253T>C and m.13528A>G) occurred in highly variable aligned codon positions. These findings indicated that they must have been someway compatible with adequate fitness in our congeneric extinct relatives.

It must be noted that the human reference sequence corresponds to a single individual of European descent with some rare polymorphisms [80]. It is likely that the reference sequences of non-human primate species might contain nucleotide variants that do not code for the most common amino acids in these species but population data on their mitochondrial genome are not presently available. However, a phylogenetic approach might indicate the persistence of variants along phyletic diversification, suggesting that they were probably frequent in ancestral populations [81]. This was the case of most reported, disease-associated amino acid variants with counterparts in non-human primates (n = 68; 75.6%) which were found to be transmitted to more than one species.

Several methods have been used for predicting the pathogenicity and severity of mutations in humans [82–86]; some of them applied to the mitochondrial genome [13]. These frequently considered evolutionary data predicted functional properties and protein structure [20,21], although some of these reports did not include topological analyses of phylogenies like determining ancestry, number and independence of evolutionary events and their polarity. Our findings showed that several reported disease-associated mutations in humans appeared to be recurrent along primate diversification in closely related species that diverged from humans less than 80 million years ago. It is therefore likely that the reported disease-associated mutations in humans in extremely conserved aligned codon positions along primate evolution are more likely to be actually pathogenic. Fourteen associated mutations with MELAS, 13 with LHON, 9 with LD, 5 with EXIT, and 30 with other pathologies fitted this criterion that might be helpful for distinguishing effectively pathogenic mutations from polymorphisms. Similarly, 14 other reported disease-associated mutations in humans occurred in variable residue positions along primate evolution resulting in amino acid changes with similar physicochemical properties. Thirteen of these positions were found to be under negative selection with SLAC (S3 Table), suggesting that they might be critically relevant for mitochondrial function.

## Supporting information

S1 Fig Maximum likelihood phylogenetic reconstruction of primate mitogenomes. Numbers close to nodes indicate aLRT support estimates. Nodes without numbers showed aLRT estimates = 100.
(PDF)

S2 Fig Graphic representation of Information on codon positions. Analyzed aligned codon position respective to Ind1, Ind2 and Ind3 estimates, negative selection, disease-associated mutations, number of species sharing amino acids resulting from disease-associated mutations, and number of independent occurrences of amino acids resulting from disease-associated mutations.
(PDF)

S1 File Alignment of 13 concatenated mt-genes. (FAS)

S2 File Newick tree file resulting from maximum likelihood phylogenetic reconstruction of primate mitogenomes. (TRE)

S1 Table Genbank accession of mitogenomes and list of species. (XLSX)

S2 Table List of independent events of codon indels in mt-genes along primate diversification. (XLSX)

S3 Table Information on codon positions. List of aligned codon positions with respective estimates of Ind1, Ind2, Ind3 and SLAC, disease-associated mutations and characteristics across primate taxa.
(XLSX)
