# Supplementary figures and images for: Disease-associated mitochondrial mutations and the evolution of primate mitogenomes
Source: PLoS One. 2017 May 16;12(5):e0177403. doi: 10.1371/journal.pone.0177403 (PMC5433710; doi:10.1371/journal.pone.0177403)

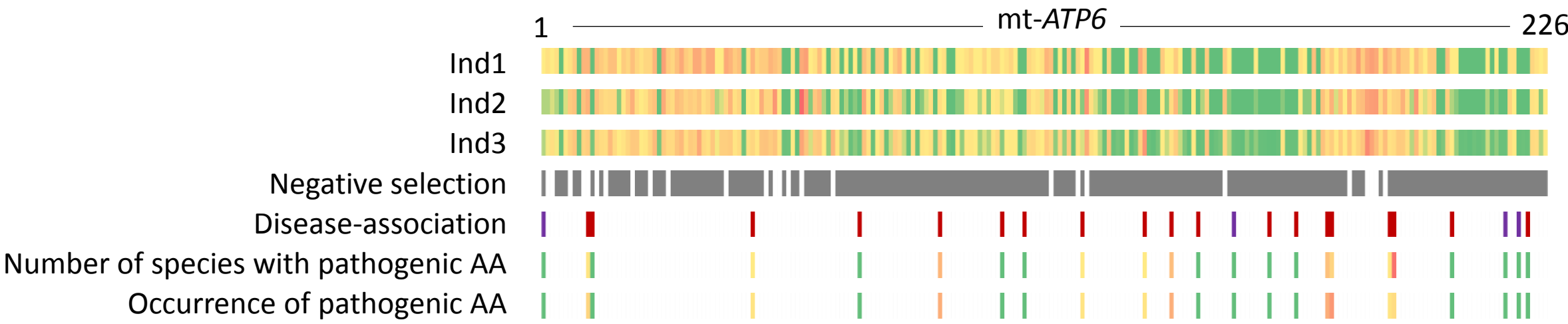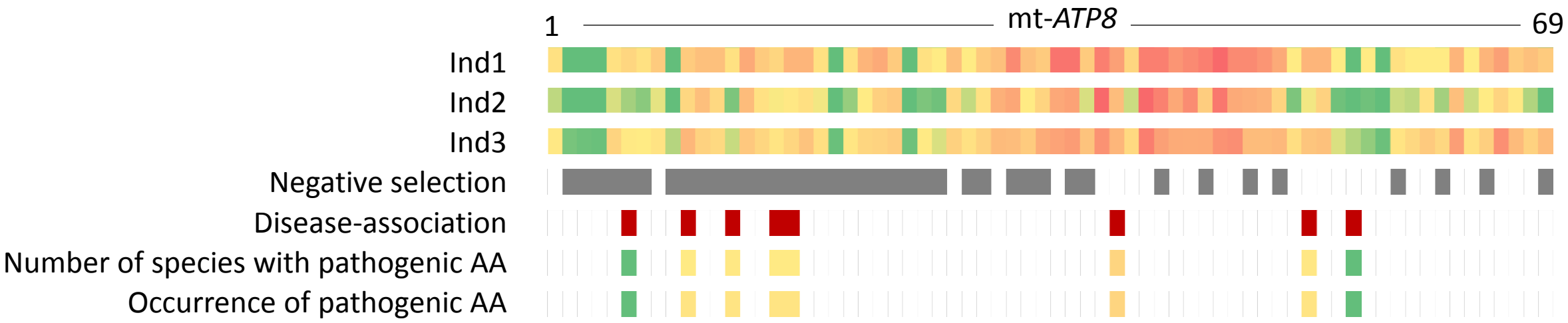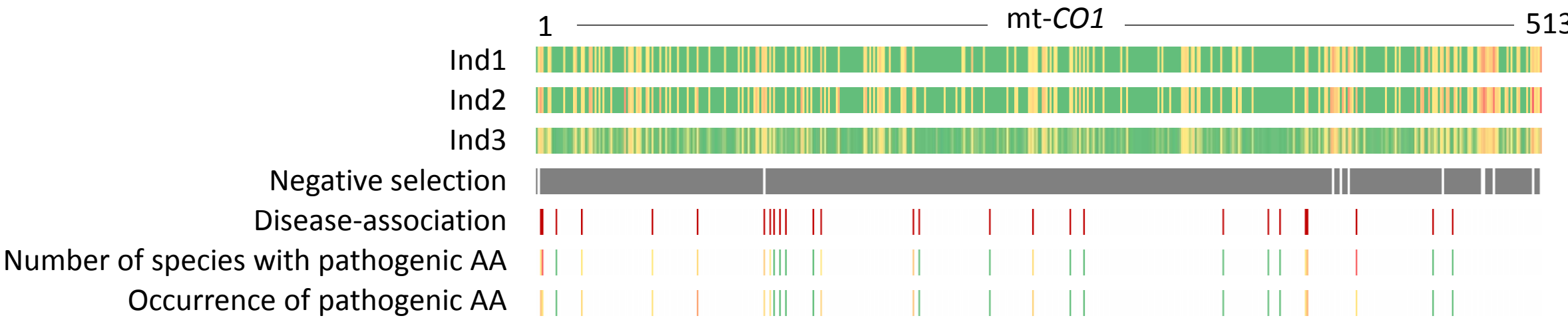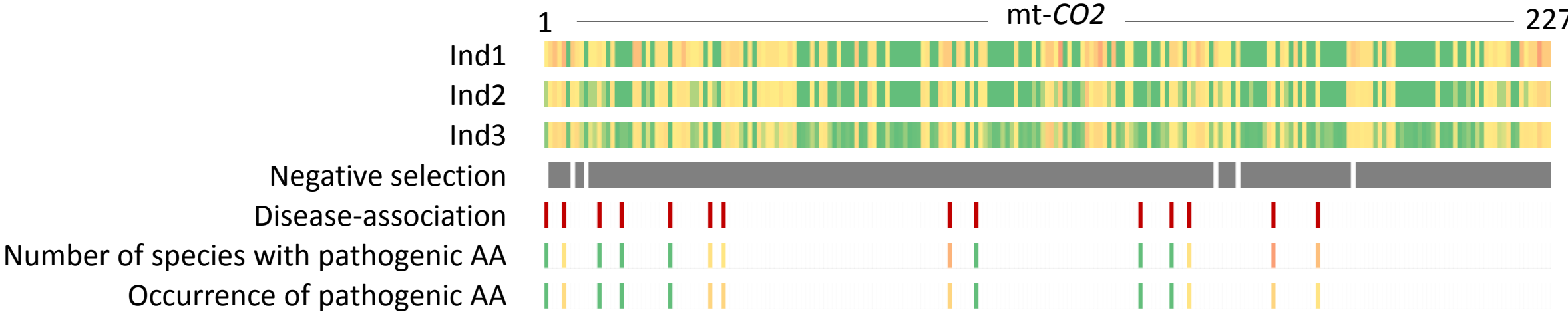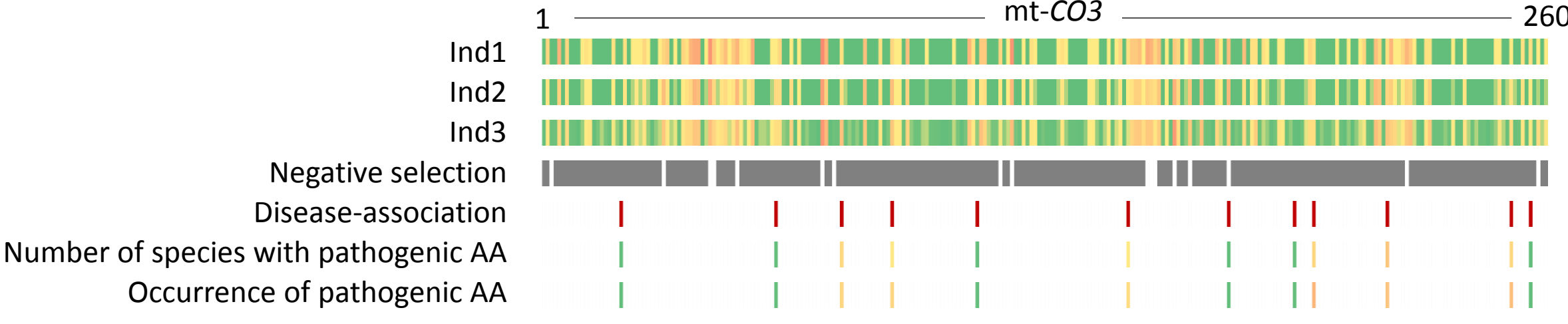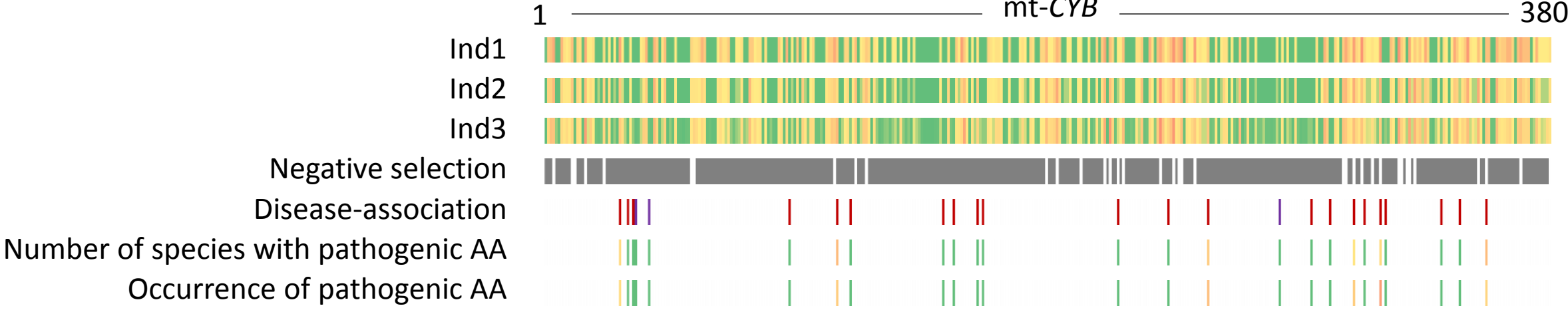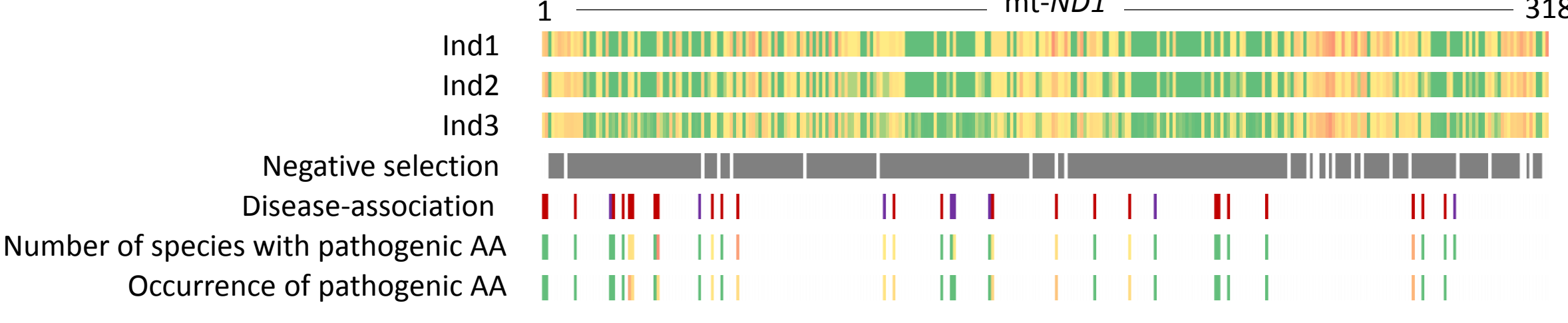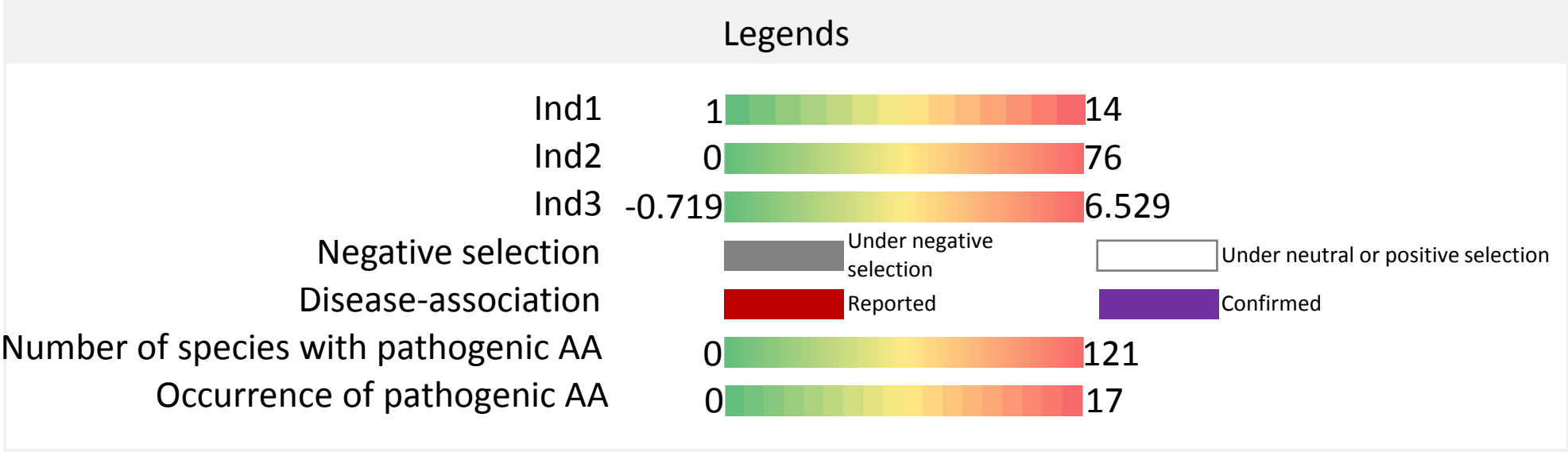

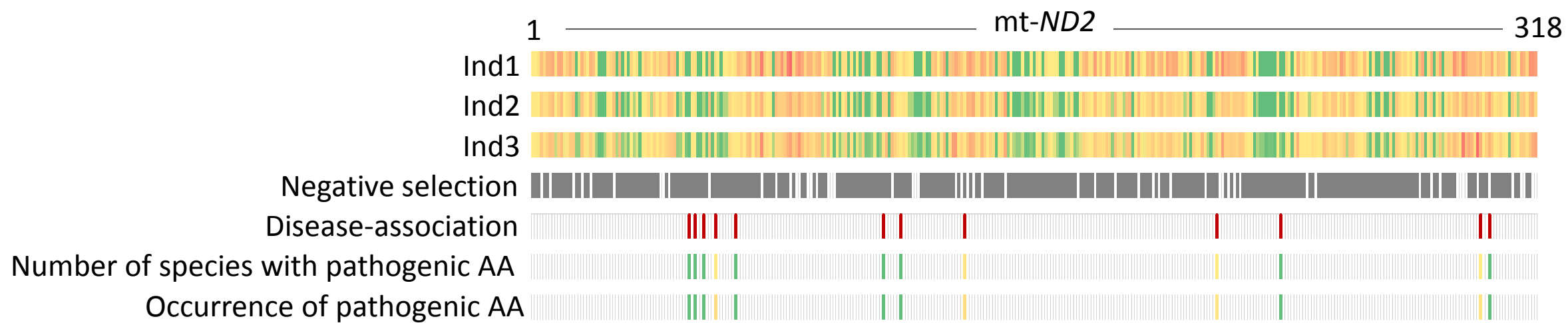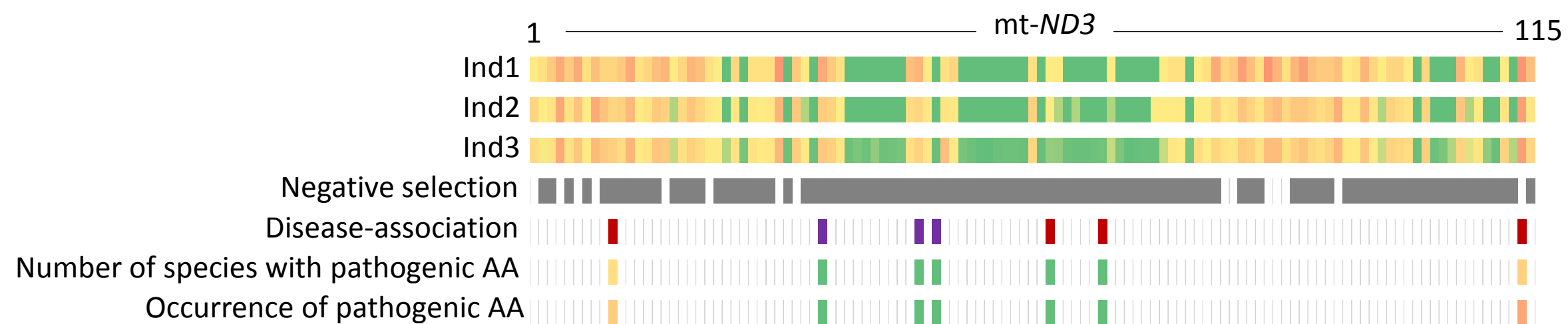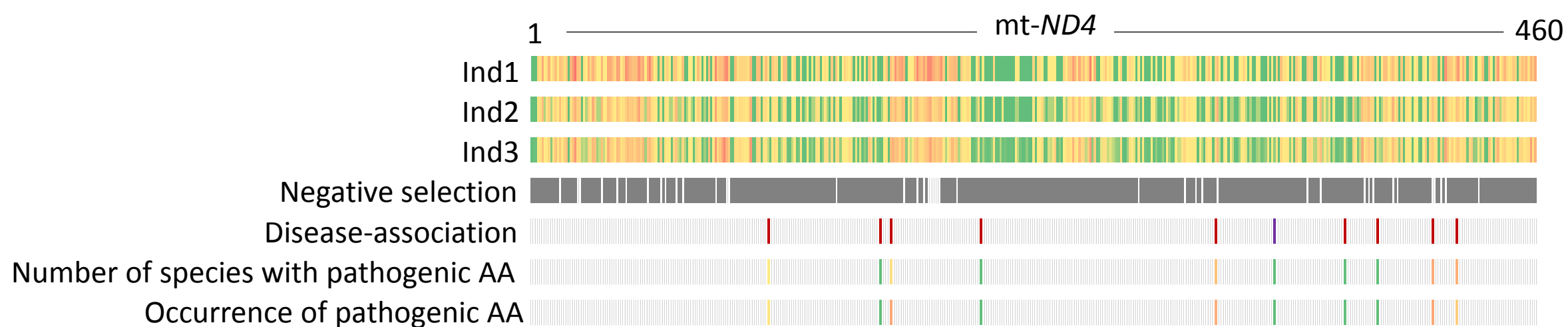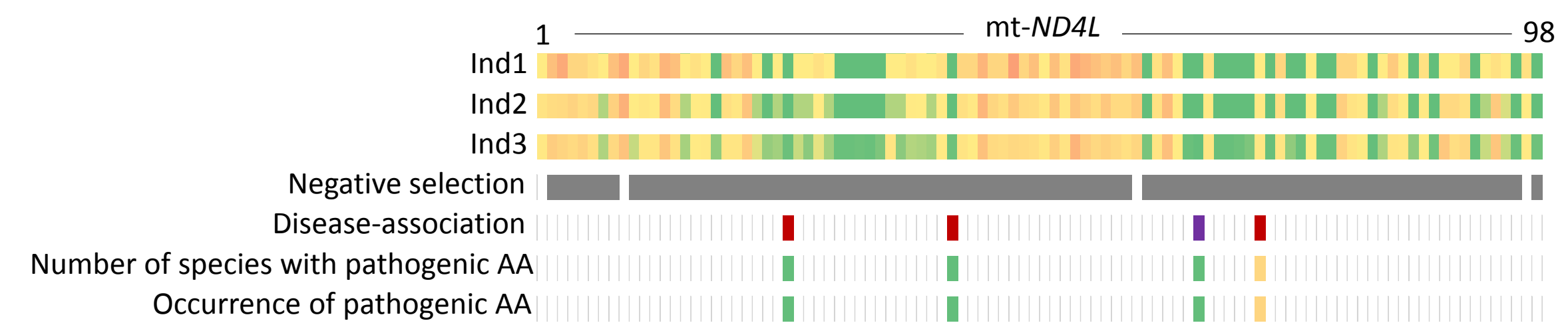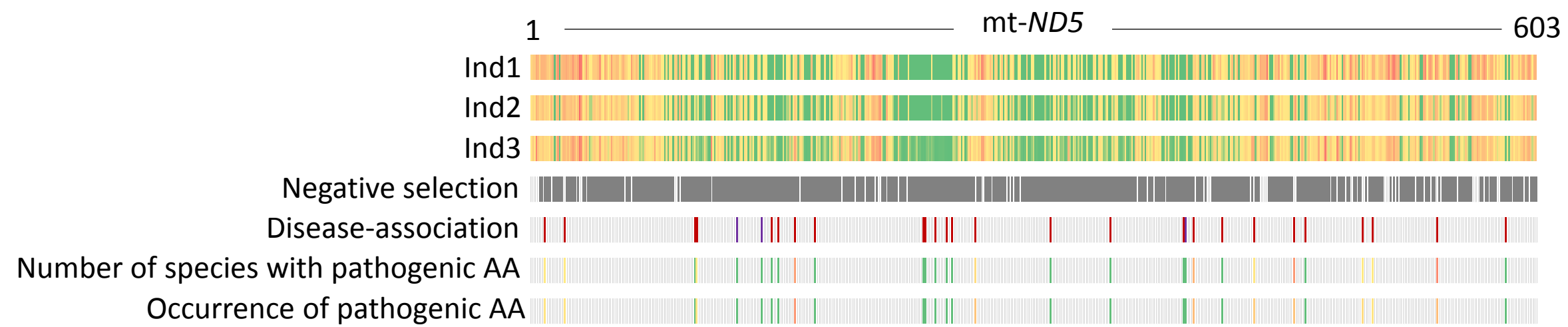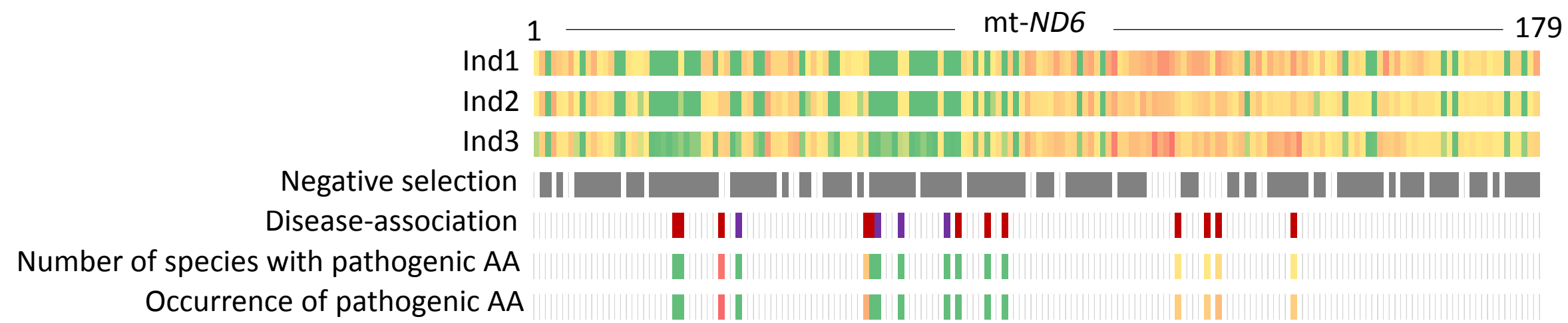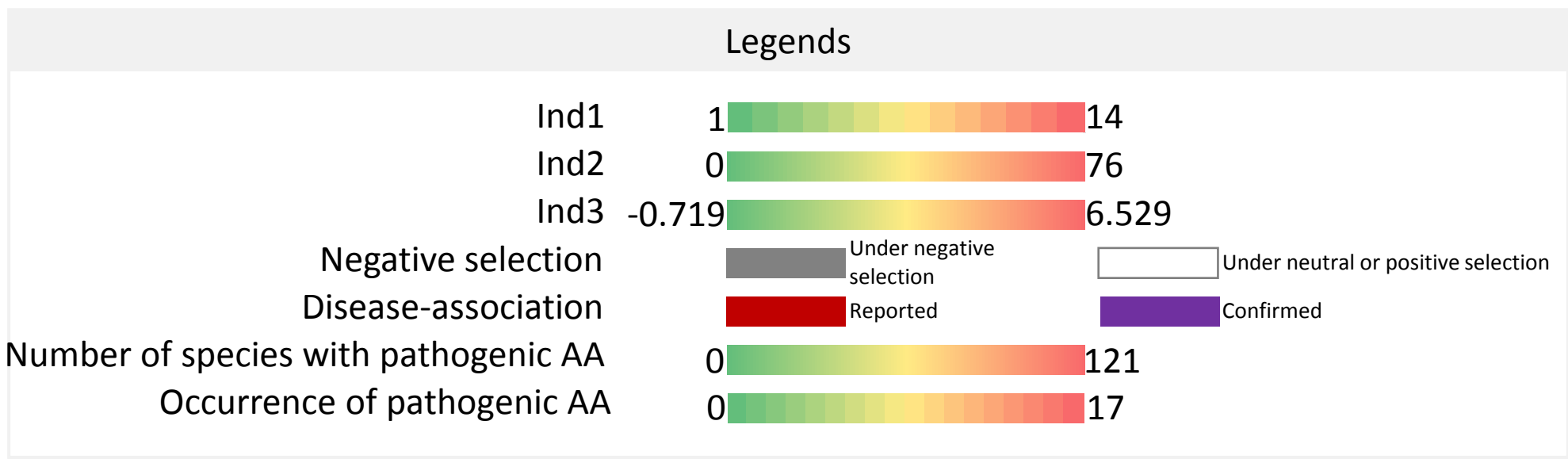

Supplement: S2 Fig — Analyzed aligned codon position respective to Ind1, Ind2 and Ind3 estimates, negative selection, disease-associated mutations, number of species sharing amino acids resulting from disease-associated mutations, and number of independent occurrences of amino acids resulting from disease-associated mutations. (PDF) [file pone.0177403.s002.pdf]
