# Supplementary material for: Disease-associated mitochondrial mutations and the evolution of primate mitogenomes
Source: PLoS One. 2017 May 16;12(5):e0177403. doi: 10.1371/journal.pone.0177403 (PMC5433710; doi:10.1371/journal.pone.0177403)
Supplement: S1 Fig — Numbers close to nodes indicate aLRT support estimates. Nodes without numbers showed aLRT estimates = 100. (PDF) [file pone.0177403.s001.pdf]

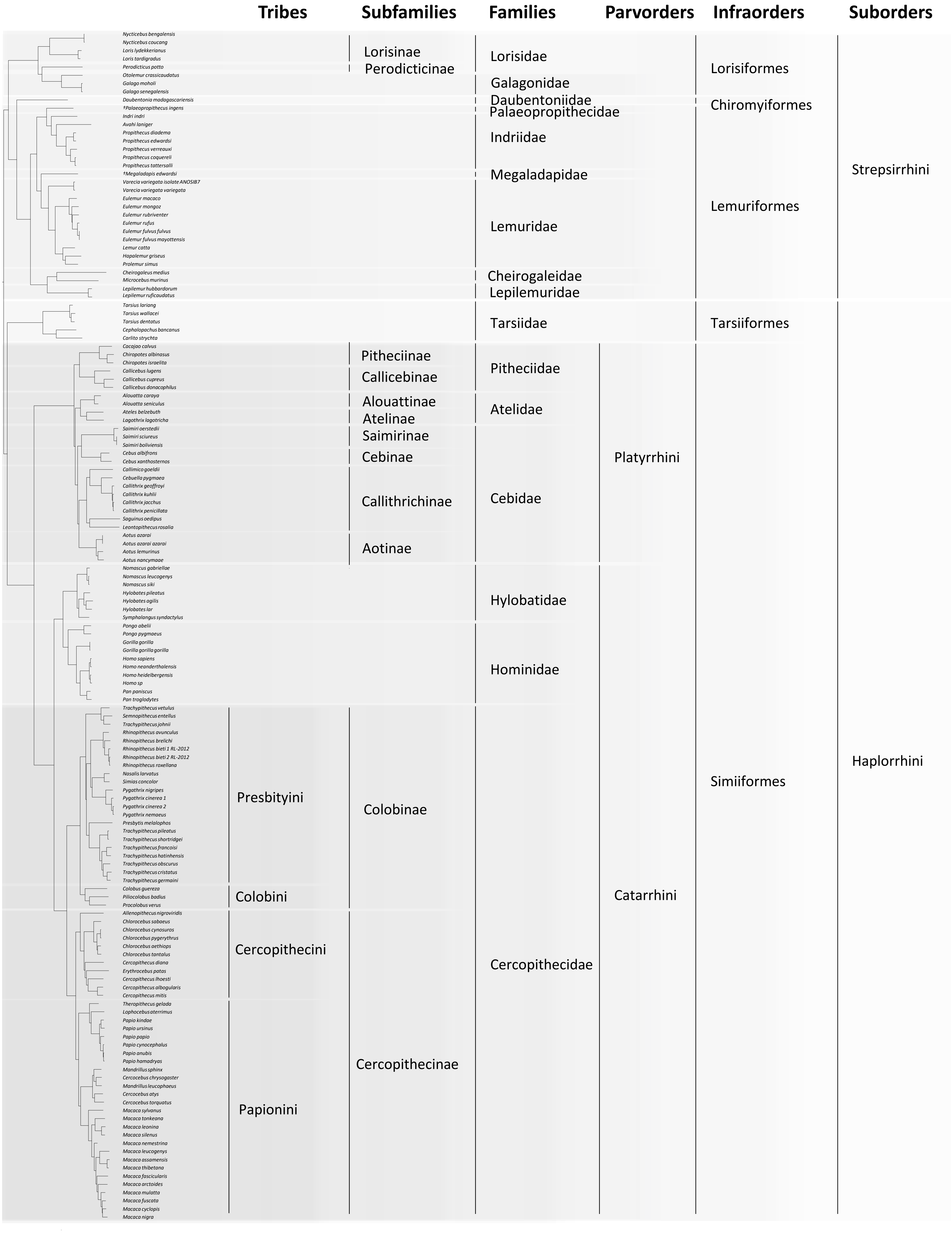
Tribes
Subfamilies
Families
Parvorders
Infraorders
Suborders
Nycticebus bengalensis
Nycticebus coucang
Loris lydekkerianus
Loris tardigradus
Perodicticus potto
Otolemur crassicaudatus
Galago moholi
Galago senegalensis
Daubentonia madagascariensis
†Palaeopropithecus ingens
Indri indri
Avahi laniger
Propithecus diadema
Propithecus edwardsi
Propithecus verreauxi
Propithecus coquereli
Propithecus tattersalli
†Megaladapis edwardsi
Varecia variegata isolate ANOSIB7
Varecia variegata variegata
Eulemur macaco
Eulemur mongoz
Eulemur rubriventer
Eulemur rufus
Eulemur fulvus fulvus
Eulemur fulvus mayottensis
Lemur catta
Hapalemur griseus
Prolemur simus
Cheirogaleus medius
Microcebus murinus
Lepilemur hubbardorum
Lepilemur ruficaudatus
Tarsius lariang
Tarsius wallacei
Tarsius dentatus
Cephalopachus bancanus
Carlito strychta
Cacajao calvus
Chiropotes albinasus
Chiropotes israelita
Callicebus lugens
Callicebus cupreus
Callicebus donacophilus
Alouatta caraya
Alouatta seniculus
Ateles belzebuth
Lagothrix lagotricha
Saimiri oerstedii
Saimiri sciureus
Saimiri boliviensis
Cebus albifrons
Cebus xanthosternos
Callimico goeldii
Cebuella pygmaea
Callithrix geoffroyi
Callithrix kuhlii
Callithrix jacchus
Callithrix penicillata
Saguinus oedipus
Leontopithecus rosalia
Aotus azarai
Aotus azarai azarai
Aotus lemurinus
Aotus nancymaae
Nomascus gabriellae
Nomascus leucogenys
Nomascus siki
Hylobates pileatus
Hylobates agilis
Hylobates lar
Symphalangus syndactylus
Pongo abelii
Pongo pygmaeus
Gorilla gorilla
Gorilla gorilla gorilla
Homo sapiens
Homo neanderthalensis
Homo heidelbergensis
Homo sp
Pan paniscus
Pan troglodytes
Trachypithecus vetulus
Semnopithecus entellus
Trachypithecus johnii
Rhinopithecus avunculus
Rhinopithecus brelichi
Rhinopithecus bieti 1 RL-2012
Rhinopithecus bieti 2 RL-2012
Rhinopithecus roxellana
Nasalis larvatus
Simias concolor
Pygathrix nigripes
Pygathrix cinerea 1
Pygathrix cinerea 2
Pygathrix nemaeus
Presbytis melalophos
Trachypithecus pileatus
Trachypithecus shortridgei
Trachypithecus francoisi
Trachypithecus hatinhensis
Trachypithecus obscurus
Trachypithecus cristatus
Trachypithecus germaini
Colobus guereza
Piliocolobus badius
Procolobus verus
Allenopithecus nigroviridis
Chlorocebus sabaeus
Chlorocebus cynosuros
Chlorocebus pygerythrus
Chlorocebus aethiops
Chlorocebus tantalus
Cercopithecus diana
Erythrocebus patas
Cercopithecus lhoesti
Cercopithecus albogularis
Cercopithecus mitis
Theropithecus gelada
Lophocebus aterrimus
Papio kindae
Papio ursinus
Papio papio
Papio cynocephalus
Papio anubis
Papio hamadryas
Mandrillus sphinx
Cercocebus chrysogaster
Mandrillus leucophaeus
Cercocebus atys
Cercocebus torquatus
Macaca sylvanus
Macaca tonkeana
Macaca leonina
Macaca silenus
Macaca nemestrina
Macaca leucogenys
Macaca assamensis
Macaca thibetana
Macaca fascicularis
Macaca arctoides
Macaca mulatta
Macaca fuscata
Macaca cyclopis
Macaca nigra
Presbityini
Colobini
Cercopithecini
Papionini
Lorisinae
Perodicticinae
Pitheciinae
Callicebinae
Alouattinae
Atelinae
Saimirinae
Cebinae
Callithrichinae
Aotinae
Colobinae
Cercopithecinae
Lorisidae
Galagonidae
Daubentoniidae
Palaeopropithecidae
Indriidae
Megaladapidae
Lemuridae
Cheirogaleidae
Lepilemuridae
Tarsiidae
Pitheciidae
Atelidae
Cebidae
Hylobatidae
Hominidae
Cercopithecidae
Platyrrhini
Catarrhini
Lorisiformes
Chiromyiformes
Lemuriformes
Tarsiiformes
Simiiformes
Strepsirrhini
Haplorrhini
